# 

**Published:** 1813-10

**Authors:** 


### MONTHLY CATALOGUE OF MEDICAL BOOKS.

A Clinical History of Acute Rheumatism, or the Rheumatic Fever. II. A Clinical History of the Nodosity of the Joints. By John Haygarth, M.D. 8vo.—Cadell and Co.

Particulars of the successful Treatment of a Case of Hydrophobia, with Observations. By Price Wynne. 8vo.—Longman and Co.

The Report of the London Committee of associated Apothecaries and Surgeon-Apothecaries of England and Wales, with the Resolutions proposed as the Bases of a new Bill. 8vo.—Callow.

Treatise on the History, Nature, and Treatment, of Chincough; including a variety of Cases and Dissections: to which is subjoined, An Inquiry into the relative Mortality of the principal Diseases of Children. By R. Watt, M.D. 8vo.—Longman and Co.

A Catalogue of Medical Books, containing the most modern and approved Works on Anatomy, Medicine, Surgery, Midwifery, Materia Medica, Chemistry, Veterinary Surgery, &c. &c. Corrected to October 1, 1813.—Highley and Son.

Anatomical Examinations.—A complete Series of Anatomical Questions, with Answers; the Answers arranged so as to form an Elementary System of Anatomy, and intended as preparatory to Examinations at Surgeons' Hall. To which are annexed, Tables of the Bones, Muscles, and Arteries. Second Edition; 2 vols. small 8vo. —Highley and Son.

Cullen's Nosology, or a Systematic Arrangement of Diseases into Classes, Orders, Genera, and Species; with accurate Definitions. (Translated from the Latin.) Foolscap 8vo —Highley and Son.

A Medical Dictionary, containing an Explanation of the Terms in Surgery, Medicine, Midwifery, Anatomy, Chemistry, &c. &c. By John James Watt. Second Edition; foolscap 8vo.—Highley and Son.

A Compendium of Anatomy, or a concise and clear Description of the Human Body; with the Physiology or Natural History of the various Actions of its different Organs and Parts. Illustrated with engravings. Containing also an Essay on Suspended Animation, with the proper means to be used for the Recovery of Drowned Persons. By William Burke, Surgeon. Second Edition. 12mo.—Highley and Son.

---

### *NOTICES TO CORRESPONDENTS.*

*Mr. Gibbons' interesting case of the imposture of Anne Foulkes, will be inserted in our next Number; also communications from Dr. Sutton; Mr. James Atkinson; Mr. J. Webster; &c. &c.*

*Mr Jones is informed that the letter signed Dr. Douglas, of Manchester, though ingeniously written, was suspected to be spurious, and was therefore not admitted; it has been mislaid, or it would have been returned as requested.*

*The Editors some time ago engaged that a General Index to the Medical and Physical Journal should be given at the end of the 30th Volume. A single Correspondent has reminded them of that promise. They accordingly acquainted the Proprietors with the circumstance, and they have agreed, that as soon as five hundred Subscribers shall have sent their names as purchasers of the Index, to the publisher, at No. 1, Paternoster-row, the work shall be proceeded upon without delay. But the requisite expence is too great to be risked without such subscription.*

